# XComposition: multimodal deep learning model to measure body composition using chest radiographs and clinical data

**DOI:** 10.1093/radadv/umaf035

**Published:** 2025-10-03

**Authors:** Ehsan Alipour, Samuel Gratzl, Ahmad Algohary, Hao Lin, Manoj Bapat, Duy Do, Charlotte Baker, Tricia Rodriguez, Brianna M Goodwin Cartwright, Jennifer Hadlock, Peter Tarczy-Hornoch, Anand Oka, Nicholas Stucky

**Affiliations:** Truveta Inc, Bellevue, WA 98004, United States; Department of Biomedical and Health Informatics, University of Washington, Seattle, WA 98195, United States; Truveta Inc, Bellevue, WA 98004, United States; Truveta Inc, Bellevue, WA 98004, United States; Truveta Inc, Bellevue, WA 98004, United States; Truveta Inc, Bellevue, WA 98004, United States; Truveta Inc, Bellevue, WA 98004, United States; Truveta Inc, Bellevue, WA 98004, United States; Truveta Inc, Bellevue, WA 98004, United States; Truveta Inc, Bellevue, WA 98004, United States; Department of Biomedical and Health Informatics, University of Washington, Seattle, WA 98195, United States; Institute for Systems Biology, Seattle, WA 98109, United States; Department of Biomedical and Health Informatics, University of Washington, Seattle, WA 98195, United States; Truveta Inc, Bellevue, WA 98004, United States; Truveta Inc, Bellevue, WA 98004, United States

**Keywords:** body composition, chest radiographs, multimodal data fusion, deep learning

## Abstract

**Background:**

Body composition metrics such as visceral fat volume, subcutaneous fat volume, and skeletal muscle volume are important predictors of cardiovascular disease, diabetes, and cancer prognosis.

**Purpose:**

We explore the use of deep learning to estimate body composition metrics from chest radiographs and a small set of easily obtainable clinical variables.

**Materials and Methods:**

A retrospective cohort of patients with concurrent noncontrast abdominal CT’s and frontal chest radiographs within 3 months of each other was selected. A multitask, multimodal, deep learning model using chest radiographs and clinical variables (age, sex at birth, height and weight extracted from electronic medical records) was trained to estimate the body composition metrics. Reference standard was body composition, including subcutaneous fat volume, measured on CT.

**Results:**

Our final cohort consisted of 1118 patients (582 female and 538 male subjects) from 30 health systems across the United States with imaging performed from 2010 to 2024. The mean age at imaging was 67 years (SD: 17), mean height was 1.67 meters (SD: 0.2), and mean weight was 78 kg (SD: 20). Average values for visceral fat, subcutaneous fat, and skeletal muscle indices were 59.39 cm^2^/m^2^ (SD: 39.26), 88.13 cm^2^/m^2^ (SD: 58.52), and 44.81 cm^2^/m^2^ (SD: 15.49). The best-performing model achieved a Pearson correlation of 0.85 (95% CI: 0.81–0.88) for subcutaneous fat volume, 0.76 (0.65–0.80) for visceral fat volume, and 0.58 (0.49–0.67) for skeletal muscle volume with the multimodal model outperforming unimodal models (*P* = .0001 for subcutaneous fat volume). Mean absolute errors of the best performing models for subcutaneous and visceral fat volumes were 1054 cm^3^/m^2^ and 667 cm^3^/m^2^, respectively.

**Conclusion:**

We introduced a multimodal deep learning model leveraging chest radiographs to estimate body composition. Our model can facilitate large-scale studies by estimating body composition using a chest radiograph and commonly available clinical variables.

**Abbreviations** 

BMI = body mass index; CNN = convolutional neural network; MAE = mean absolute error

**Summary** 

We developed a multimodal deep learning model using a chest radiograph and 4 clinical variables to estimate body composition including subcutaneous adipose and visceral adipose tissues measured on CT.

**Key Results** 

- A multimodal deep learning model based on chest radiographs and clinical data can estimate body composition metrics like subcutaneous (Pearson’s *R*: 0.85) and visceral fat volume (Pearson’s *R*: 0.76).
- The late fusion strategy performed best in estimating body composition metrics when combining imaging and clinical data (*P* < .04 for subcutaneous fat volume).
- The multimodal model outperforms both imaging-only model and clinical-only model in estimating body composition metrics (*P* < .001 for subcutaneous fat volume).

## Introduction

Body composition metrics have attracted significant attention as predictors of health-related outcomes. Multiple studies have demonstrated correlations between body composition metrics like visceral fat area and skeletal muscle index and chronic noncommunicable disease, including cardiovascular disease and diabetes.^1^^,^^2^ In addition, studies have found that abnormal body composition measures can be a predictor of poor prognosis in patients with cancer, including those with breast and colorectal cancers.^3^ These correlations are often stronger than the correlation between these diseases and commonly used body composition surrogates like body mass index (BMI) and weight.^1^^,^^2^^,^^4^ Measures such as BMI fail to capture critical information about body composition, including muscle mass, visceral adipose tissue (which has been shown to be associated with pathologic conditions^5^), and subcutaneous adipose tissue. Studies have also explored the use of body composition metrics for opportunistic screening of various diseases, including cardiometabolic disease, osteoporosis, and steatohepatitis.^6^

Common methods for calculating body composition metrics include whole-body magnetic resonance, CT imaging, and bioelectrical impedance analysis.^7^ Scientists have validated using body composition metrics calculated from 2-dimensional slices in the L3 section of abdominal CT scans as surrogates of whole-body composition metrics.^8^^,^^9^ However, these methods may not be available for all patients because of limited resources, risk of radiation exposure, or need for specialized equipment, software, and staff. Hence, new strategies to estimate body composition measures using readily accessible clinical and imaging data would enable the calculation of body composition metrics for a larger pool of individuals. One avenue is to use simpler modalities like chest radiographs to estimate body composition. A recent study on opportunistic screening of type 2 diabetes demonstrated that chest radiographs encode information about fat distribution that can be used to screen for type 2 diabetes.^10^

Multimodal machine learning approaches in medicine have introduced novel opportunities to develop deep learning models that use a variety of data sources for predictive tasks. The use of multimodal data can improve predictive performance. The process of combining multiple data modalities is called data fusion. Three main approaches exist for data fusion in machine learning, including early (feature level), intermediate, and late (decision level) fusion. Although the choice of data fusion can have a significant impact on model performance, fusion is often underexplored in biomedical literature.

In this study, we aim to create a multimodal deep learning model to estimate CT-based body composition metrics by combining readily available clinical data with chest radiographs. Such a model could be applied with minimal cost retrospectively or prospectively on any person with a chest radiograph, enabling greater use of body composition metrics in research and clinical care, including screening. In addition, we experimented with different ways of combining the clinical data and imaging data to identify the best fusion strategy for this task.

## Methods

### Dataset

The clinical and imaging data used in our study were compiled from the Truveta Data.^11^^,^^12^ Truveta provides access to continuously updated and linked electronic health records data from 30 health systems across the United States.^13^ It consisted of deidentified records from a subset of the Truveta Data, which also contributed imaging data. Truveta Data used in this study was accessed on July 14, 2024. This study performs an analysis of deidentified electronic health records data accessed via Truveta Studio. Deidentification is attested to through expert determination in accordance with the HIPAA Privacy Rule. This study used only deidentified patient records and therefore did not require institutional review voard approval. Refer to the Supplementary Material Section 1 for additional details.

**Figure 1.**
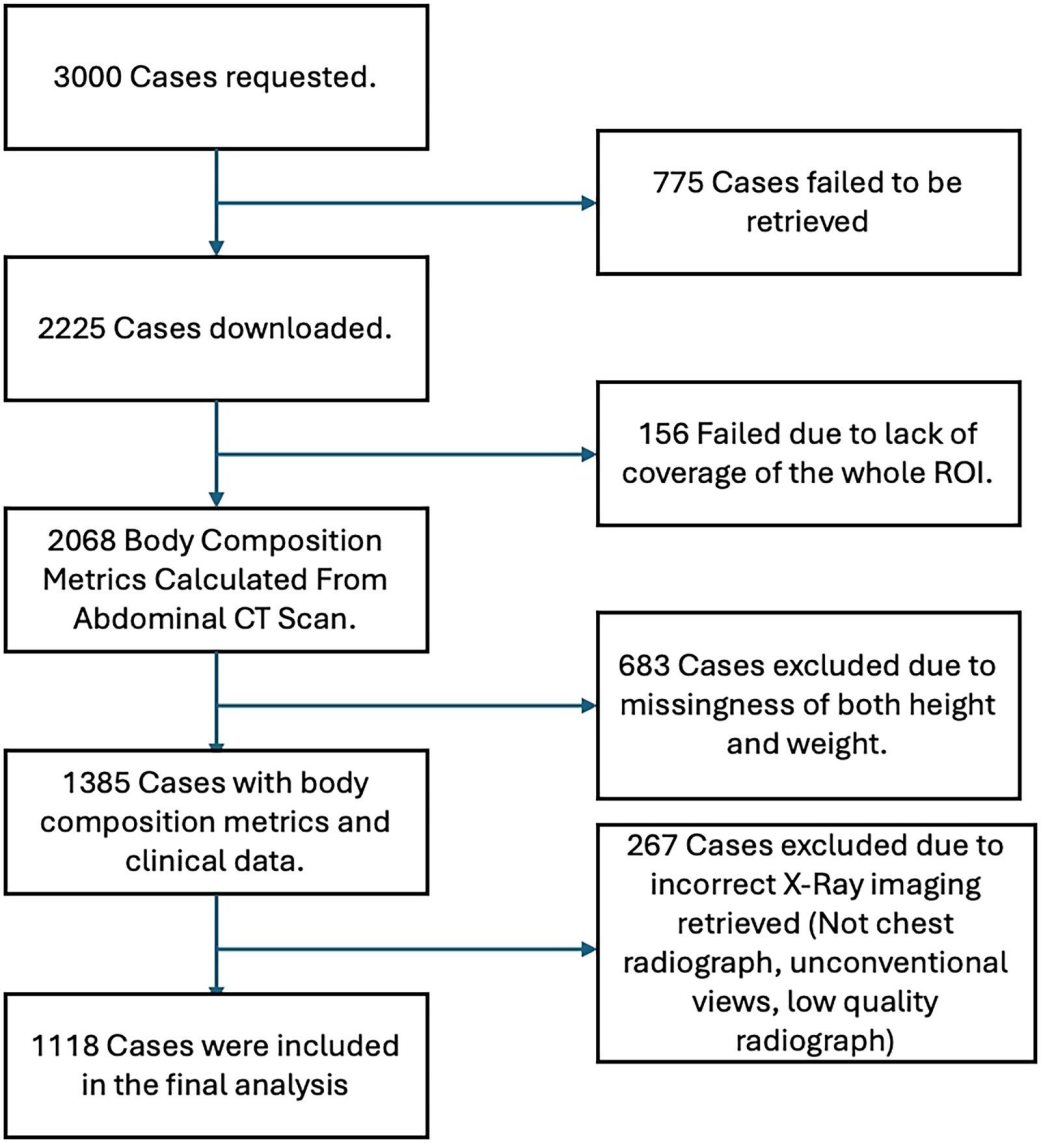
Cohort flowchart. We initially request 3000 cases. Of these, 1118 cases met all our inclusion criteria and were included in our study. Individuals with missing clinical data were excluded if their age or sex was missing or if both height and weight were missing. Inaccurate clinical data includes height and weight measurements outside acceptable ranges (height 100–280 cm, weight: 30–600 kg). ROI = region of interest.

Using Truveta Data, we identified adult individuals who had a chest radiograph within 3 months of an abdominal CT scan without any contrast agent. We further limited our inclusion criteria to patients with a weight measurement within 1 month of the chest radiograph and had a height measurement anytime in their adult life. Although our search returned 312 444 unique cases, because of computational constraints, we randomly sampled 3000 patients. The imaging data, in addition to the age at the time of imaging, sex at birth, weight, and height, were collected for every patient. The cohort flowchart diagram can be seen in Figure 1. Data characteristics, including age, sex at birth, and body composition distributions, are reported in Table 1.

**Table 1.** Population characteristics and volume based body composition distribution across the training, validation and test sets.

		Train (*n* = 726)		Validation (*n* = 177)		Test (*n* = 215)		All (*n* = 1118)		Significance
		Mean	SD	Mean	SD	Mean	SD	Mean	SD	*P* value
Variable	Category									
Age		67.13	17.36	68.15	16.31	66.13	16.8	67.1	17.09	.51
Height (m)		1.69	0.18	1.66	0.16	1.7	0.27	1.69	0.2	0.12
Weight (kg)		77.34	19.66	79.66	22.11	77.74	20.28	77.78	20.18	.39
		Count	%	Count	%	Count	%	Count	%	
Sex	Female	374	51.52	90	50.85	118	54.88	582	52.06	.64
	Male	352	48.48	87	49.15	97	45.12	536	47.94	
		Mean	SD	Mean	SD	Mean	SD	Mean	SD	*P* value
Skeletal muscle volume (cm^3^)		2241.42	783.94	2265.02	848.56	2233.83	843.84	2243.69	805.52	.92
Visceral fat volume (cm^3^)		2850.55	1940.01	2877.18	1734.09	2791.08	1916.73	2843.33	1902.95	.89
Subcutaneous fat volume (cm^3^)		4464.93	2832.39	4789.71	2782.95	4560.83	2700.3	4534.79	2799.63	.38
Vertebral bone volume (cm^3^)		347.09	76.06	347.6	85.02	342.74	73.76	346.34	77.06	.75
Fat free volume (cm^3^)		7982.08	2166.6	8249.24	2807.68	8137.64	2421.02	8054.29	2328.64	.33
Intramuscular fat (cm^3^)		220.17	167.36	213.24	156.64	224.87	175.39	219.98	167.18	.79
Vertebral bone density (HU)		320.87	78.46	322.87	71.53	329.11	86.03	322.77	78.93	.4
Muscle radiodensity (HU)		19.49	15.4	19.52	13.29	19.23	15.14	19.44	15.02	.97
Aortic calcification score		8.67	14.58	9.22	13.97	8.72	12.55	8.77	14.11	.9
Number of plaques in abdominal aorta		10.62	11.16	10.17	10.67	10.64	11.06	10.55	11.05	.88
Skeletal muscle index		792.43	276.83	828.56	326.89	788.1	303.19	797.32	290.48	.29
Subcutaneous fat index		1620.39	1099.3	1803.23	1198.27	1636.58	1013.87	1652.45	1100.84	.14
Visceral fat index		999.76	667.44	1038.11	588.53	984.25	664.47	1002.85	654.65	.7
Fat free index		2830.93	765.86	3026.78	1080.12	2888.8	906.78	2873.07	852.64	.02

L3-based body composition distribution is available in the appendix.

HU = Hounsfield units.

### Imaging characteristics

Noncontrast abdominal CT scans acquired for routine clinical practice across the health care organizations represented in Truveta Data across the United States between 2010 and 2024 were used. All included scans encompassed the abdomen from T12 to L5 vertebral levels, and acquisition protocols varied by the institution and clinical indication for the imaging. Slice thickness varied from 0.6 mm to 5 mm, and tube voltage was typically 120 kVp. Scanner type included various models from GE Healthcare, Siemens Healthineers, and Philips Healthcare systems. Both helical and axial acquisition modes were included.

### Missing data

For cases where the recorded height or weight was outside of the acceptable range or missing, we imputed the missing value using linear regression on the rest of the variables.

### Body composition metrics calculation

The ground truth values for body composition were calculated using a combination of 2-dimensional slices from the T12 vertebrae to the L5 vertebrae for volumetric body composition metrics and on the mid-L3 level for the single-slice body composition metrics in the abdominal CT scans. The TotalSegmentator^14^ tool was used to automatically segment out the various body composition sections in CT scans and identify the T12, L3, and L5 levels. The list of body composition metrics calculated can be found in Table 1 (for L3-level body composition metrics, refer to Table S1 in the appendix.). An in-house rule-based Python script based on available literature was used to calculate the body composition metrics from the segmentation masks.^15–17^ The deep learning–generated segmentation masks were visually inspected to ensure accuracy. More details can be found in the Supplementary Material Section 2.

### Chest radiograph preprocessing

All retrieved radiographs were manually reviewed by a physician scientist to ensure only posteroanterior or anteroposterior chest radiographs are included, and images contain no significant artifacts. Chest radiograph pixel values were normalized using *z*-score normalization. All x-rays were resized to 512 × 512 pixels. *Z*-score normalization was applied to all numerical variables and all calculated body composition metrics to ensure numerical stability during training using the mean and SD of the training set. For data augmentation, we applied random rotation, random flipping, and randomly changed the contrast and noise levels in the images.

### Modeling approach and fusion strategy

The data was split with a 0.8/0.2 ratio to generate the training dataset and the hold-out test set. The test set was only used to measure the final model performance. Additionally, 20% of the training data was used as a validation set for hyperparameter tuning.

Three main model categories were developed: clinical, imaging, and multimodal models. The clinical model was developed using a shallow neural network with two fully connected layers using the 4 clinical variables (height, weight, age, and sex at birth).

The imaging model consisted of a multitask convolutional neural network (CNN). Multiple CNN architectures (ResNet10, ResNet50, ResNet101) were tested; however, ResNet18^18^ was selected based on validation performance. The parameters in the network were initiated randomly. The default ResNet18 architecture from the PyTorch (v2.4) package was used. All training was performed in a virtual machine with an NVIDIA V100 GPU with 16 GB of memory. The Adam optimizer was used for training a batch size of 16. The best performing model was trained for 80 epochs with a learning rate of 10^−5^ and weight decay of 10^−8^.

**Figure 2.**
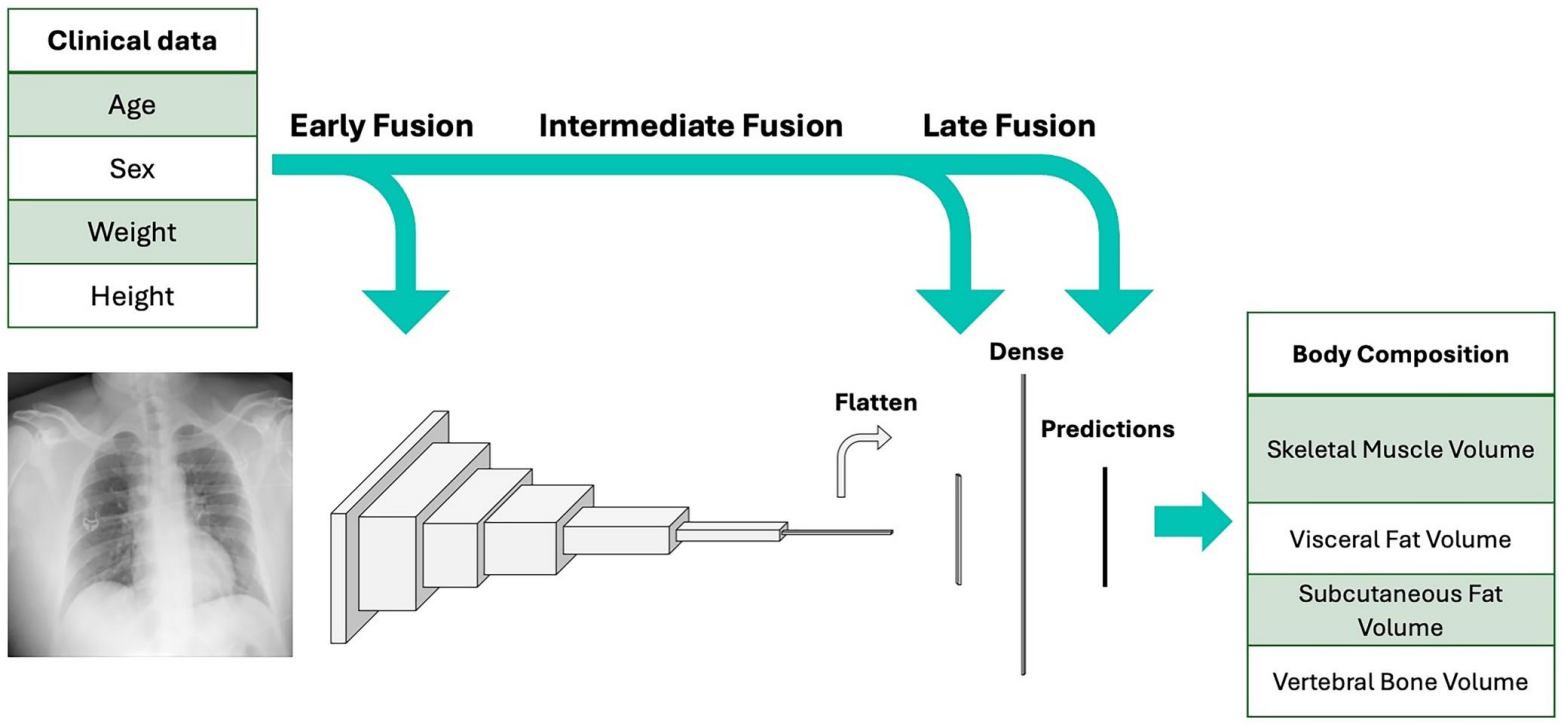
A diagram showing model architecture, input features, and the various fusion strategies used in our study. We used a resnet18 model for our convolutional neural network.

We experimented with 3 fusion strategies, comparing early, intermediate, and late fusion of the clinical and imaging data.^19^^,^^20^ Fusion strategies are depicted in Figure 2. More information can be found in the Supplementary Material Section 3.

### Evaluation

Huber loss,^21^ which is a combination of mean absolute error (MAE) and mean squared error, was used to train the models and evaluate the performance. The equation for Huber loss is described here. In the equations, y is the ground truth, $\hat{y}$ is the estimated value, and δ defines the transition point for the loss. In addition, weight decay was used for regularization. The correlation between the predicted outcomes versus the ground truth values is reported for all body composition metrics.


Huber Lossδ(y, y^)= {12 (y-y^)2 if |y- y^|<δδ(|y- y^|-12δ) Otherwise

### Explainability

Both occlusion sensitivity and integrated gradient methods were used to calculate saliency maps over all the images.^22^ Aggregate explainability maps were generated by averaging all the input images in the test set and their respective explainability maps for the 2 methods were separately examined. Refer to Supplementary Material Section 4 for fairness analysis.

### Statistical analysis

The *P* value significance level threshold was set to .05 for all analyses. Pearson correlation was calculated to compare predictions and ground truth values for all body composition metrics except aortic calcification score and number of calcified plaques. Spearman correlation was used for those due to their skewed distribution. Analysis of variance was used to identify any statistically significant difference present across the data splits for continuous variables, and the chi-square test was used for categorical variables. The Friedman test was used to identify significant differences between model performances when 3 or more models were being compared. The following cut points were defined for the correlations: poor (*r* < 0.6), moderate (0.5 ≤ *r *< 0.7), good (0.7 ≤ *r* < 0.8), very good (0.8 ≤ *r* < 0.9), and great (*r* ≥ 0.9).

### Data sharing and code availability

The data used in this study are available via a paid Truveta subscription and can be accessed at http://studio.truveta.com/. Researchers may obtain access by subscribing. Our model weights and the required code to calculate body composition metrics are shared on GitHub at https://github.com/Truveta/xcomposition_multimodal_deep_learning_body_composition. This project was done using PyTorch (version 2.4), Sci-kit Learn (version 1.5.2), and Captum (version 0.7.0).

## Results

### Study population

From a potential cohort of 3000 cases, we included a final cohort that consisted of 1118 samples. The cohort flowchart is shown in Figure 1. The average age was 68 ± 17.36 years. The average BMI was 27. Average visceral fat index was 59.39 cm^2^/m^2^ (SD: 39.26), whereas average subcutaneous fat index was 88.13 cm^2^/m^2^ (SD: 58.52) and average skeletal muscle index was 44.81 cm^2^/m^2^ (SD: 15.49). Distribution of body composition metrics can be viewed in Figure S1. There was a 20% missingness for the height measurements and 5% missingness for the weight measurements. We used imputation strategies for those missing values. The demographic distribution of the cases and distribution of the body composition metrics can be seen in Table 1.

### Clinical model performance

The clinical model included age, sex at birth, height, and weight. The clinical model achieved a good correlation of 0.77 (95% CI: 0.71–0.82) when predicting subcutaneous fat volume and index but achieved moderate to poor performance in estimating other body composition metrics. The other highest performing metrics included visceral fat area 0.69 (0.65–0.71) and vertebral bone volume with a correlation of 0.67 (0.59–0.74). More detailed performance metrics can be viewed in Table 2.

**Table 2.** Final model performance in the holdout test set comparing imaging, clinical, and multimodal models.

	Skeletal muscle volume	Visceral fat volume	Subcutaneous fat volume	Muscle radiodensity	Fat free volume
Imaging	0.45	0.7	0.78	0.61	0.47
Clinical	0.58	0.69	0.77	0.6	0.56
Multimodal	**0.58 (95% CI: 0.49–0.67)**	**0.76 (0.65–0. 80)**	**0.85 (0.81–0.88)**	**0.69 (0.61–0.75)**	**0.59 (0.50–0.67)**
*P* value	**.04**	**>.001**	**>.001**	**.02**	**>.001**
	Intramuscular fat	Aortic calcification score (Spearman)	Number of plaques in aorta (Spearman)	Skeletal muscle index	Visceral Fat index
Imaging	0.48*	0.44	0.45	0.26	0.6
Clinical	0.52	**0.69**	**0.67**	0.51	0.67
Multimodal	0.55** (0.44–0.63)***	0.66	0.68	**0.53 (0.43–0.62)**	**0.75 (0.67–0.80)**
*P* value	**>.001**	**>.001**	**>.001**	**.09**	**>.001**
	Subcutaneous fat index	Fat free index	Vertebral bone volume	Vertebral bone density	
Imaging	0.74	0.31	0.5	0.47	
Clinical	0.77	0.56	0.67	0.38	
Multimodal	**0.85 (0.80–0.88)**	**0.59 (0.49–0.67)**	**0.72 (0.65–0.78)**	**0.48 (0.36–0.57)**	
*P* value	**>.001**	**>0001**	**>.001**	**.07**	

* Pearson correlation between predictions and ground truth values reported for all models unless stated otherwise.

** Under each metric, the best performing model is shown with bold letters.

*** The 95% CI is reported for the test set.

### Imaging model performance

The image-only model outperformed the clinical model in the prediction of subcutaneous fat volume (*r* = 0.78, 0.72–0.82) and visceral fat volume (*r* = 0.70, 0.63–0.76), achieving good correlation with ground truth in both metrics. The imaging-only model also performed better in estimating muscle radiodensity and vertebral bone radiodensity compared to the clinical model.

### Combined model performance

**Figure 3.**
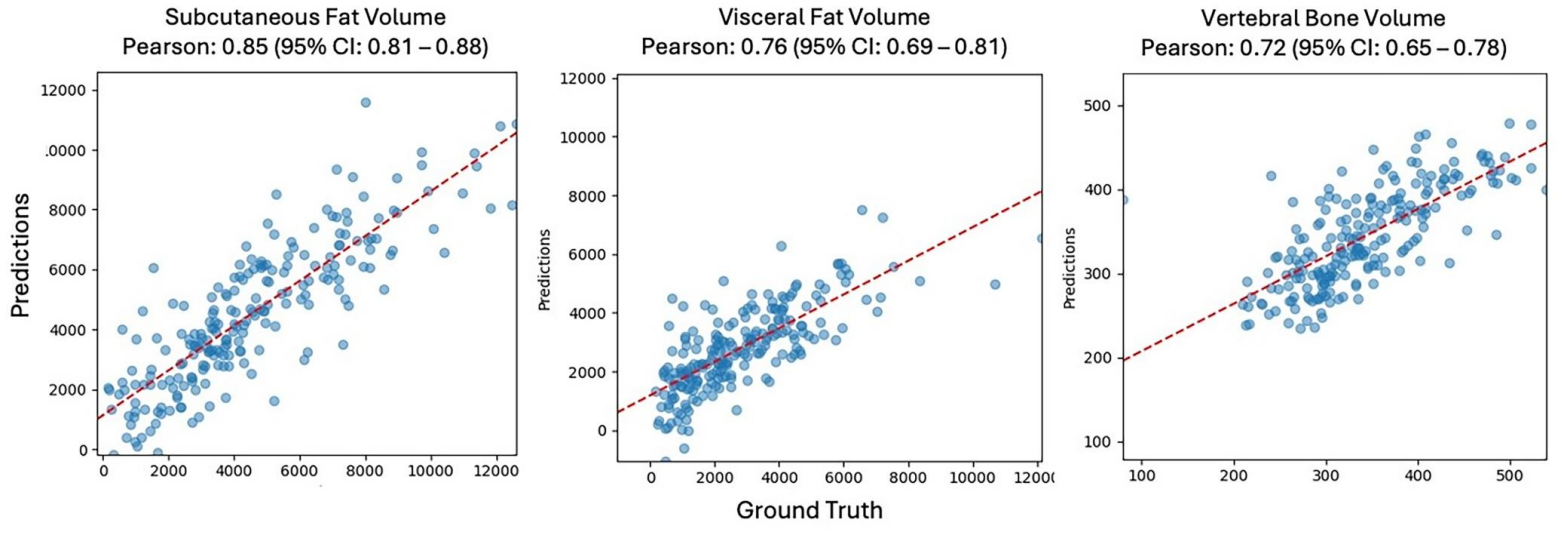
Scatter plots showing multimodal model predictions versus ground truth values (ie, values calculated from abdominal CT) for 3 of the top performing body composition metrics.

Our multimodal models achieved higher performance in estimating almost all body composition metrics, especially subcutaneous and visceral fat-related measures. Our results showed that the late fusion-based model performed best in estimating body composition metrics, followed closely by intermediate fusion. Scatter plots comparing model performance on the test set for the 3 top-performing body composition metrics are presented in Figure 3. Table 3 contains the final multimodal model performance metrics across the train, validation, and test cohorts.

**Table 3.** Final multimodal model performance across train, test and validation sets.

	Skeletal muscle volume	Visceral fat volume	Subcutaneous fat volume	Muscle radiodensity	Fat free volume
Train	0.74 (0.71–0.77)	0.76 (0.69–0.81)	0.86 (0.84–0.87)	0.74 (0.71–0.77)	0.73 (0.70–0.76)
Validation	0.74 (0.67–0.81)	0.73 (0.66–0.79)	0.82 (0.79–0.85)	0.62 (0.56–0.68)	0.63 (0.57–0.69)
Test	0.58 (0.48–0.66)	0.76 (0.69–0.81)	0.85 (0.81–0.88)	0.69 (0.61–0.75)	0.59 (0.49–0.66)
	Intramuscular fat	Aortic calcification score (Spearman)	Number of plaques in aorta (Spearman)	Skeletal muscle index	Visceral fat index
Train	0.59 (0.55–0.63)	0.70	0.70	0.70 (0.66–0.73)	0.74 (0.71–0.77)
Validation	0.45 (0.36–0.55)	0.65	0.61	0.73 (0.69–0.77)	0.71 (0.67–0.75)
Test	0.55 (0.44–0.63)	0.66	0.68	0.53 (0.43–0.62)	0.75 (0.58–0.80)
	Subcutaneous fat index	Fat free index	Vertebral bone volume	Vertebral bone density	
Train	0.85 (0.83–0.87)	0.70 (0.66–0.73)	0.76 (0.73–0.79)	0.56 (0.52–0.61)	
Validation	0.8 (0.76–0.84)	0.67 (0.60–0.74)	0.69 (0.63–0.74)	0.37 (0.25–0. 49)	
Test	0.85 (0.80–0.88)	0.59 (0.49–0.67)	0.72 (0.65–0.78)	0.48 (0.36–0.57)	

Pearson correlation is reported for all models. For aortic calcification and number of plaques, Spearman correlation is reported.

#### Early fusion

The early fusion model estimates achieved a very good correlation of 0.80 (0.75–0.84) for the subcutaneous fat index, and a good correlation for visceral fat index, visceral fat volume, and subcutaneous fat volume. The early fusion model consistently underperformed the 2 other models in estimating all body composition metrics.

#### Intermediate fusion

The intermediate fusion model achieved a correlation of 0.82 (0.78–0.86) in estimating subcutaneous fat volume and index. It also achieved good performance in estimating visceral fat volume and visceral fat index. This model outperformed the other fusion strategies in estimating muscle radiodensity (*r* = 0.69, 0.61–0.75), fat-free index (*r* = 0.60, 0.51–0.68), intramuscular fat (*r* = 0.57, 0.48–0.66), and vertebral bone radiodensity (*r* = 0.49, 0.38–0.58).

#### Late fusion

The late fusion model achieved a correlation of 0.85 (0.81–88) in estimating subcutaneous fat volume. This model also had good performance in estimating visceral fat volume (*r* = 0.76, 0.69–0.81) and vertebral bone volume (*r* = 0.72, 0.65–0.78). The Spearman correlation for skeletal muscle volume (*r* = 0.67), vertebral bone volume (*r* = 0.75), and subcutaneous fat index (*r* = 0.88) was higher than the Pearson correlations, highlighting a slightly better ability to rank observations compared to estimating their actual value. Table 4 contains performance metrics for all body composition measures across the fusion strategies.

**Table 4.** Test set performance across various fusion strategies (Pearson correlation).

	Skeletal muscle volume	Visceral fat volume	Subcutaneous fat volume	Muscle radiodensity	Fat free volume
Early	0.57 (0.47–0.65)	0.69 (0.61–0.76)	0.76 (0.69–0.81)	0.61 (0.52–0.69)	0.5 (0.39–0.59)
Intermediate	**0.58 (0.49–0.67)**	0.75 (0.68–0.80)	0.82 (0.77–0.86)	**0.69 (0.61–0.75)**	**0.59 (0.50–0.67)**
Late	**0.58 (0.48–0.66)**	**0.76 (0.69–0.81)**	**0.85 (0.81–0.88)**	0.68 (0.58–0.73)	**0.59 (0.49–0.66)**
*P* value	.15	.07	.04	.19	.01
	Intramuscular fat	Aortic calcification score (Spearman)	Number of plaques in aorta (Spearman)	Skeletal muscle index	Visceral fat index
Early	0.5 (0.39–0.60)	0.61	0.62	0.48 (0.37–0.58)	0.7 (0.62–0.76)
Intermediate	**0.57 (0.48–0.66)**	0.65	0.67	**0.53 (0.43–0.62)**	**0.75 (0.69–0.80)**
Late	0.55 (0.44–0.63)	**0.66**	**0.68**	0.51 (0.41–0.61)	**0.75 (0.58–0.80)**
	0.26	0.02	0.48	0.23	0.07
	Subcutaneous fat index	Fat free index	Vertebral bone volume	Vertebral bone density	
Early	0.8 (0.75–0.84)	0.51 (0.41–0.61)	0.69 (0.62–0.76)	0.34 (0.21–0.45)	
Intermediate	0.82 (0.78–0.86)	**0.6 (0.51–0.68)**	0.69 (0.61–0.75)	**0.49 (0.38–0.58)**	
Late	**0.85 (0.80–0.88)**	0.59 (0.49–0.67)	**0.72 (0.65–0.78)**	0.48 (0.36–0.57)	
*P* value	.15	.07	.53	.003	

**Bold** font signifies the best performing fusion strategy for each body composition metric based on the correlation coefficients.

**Figure 4.**
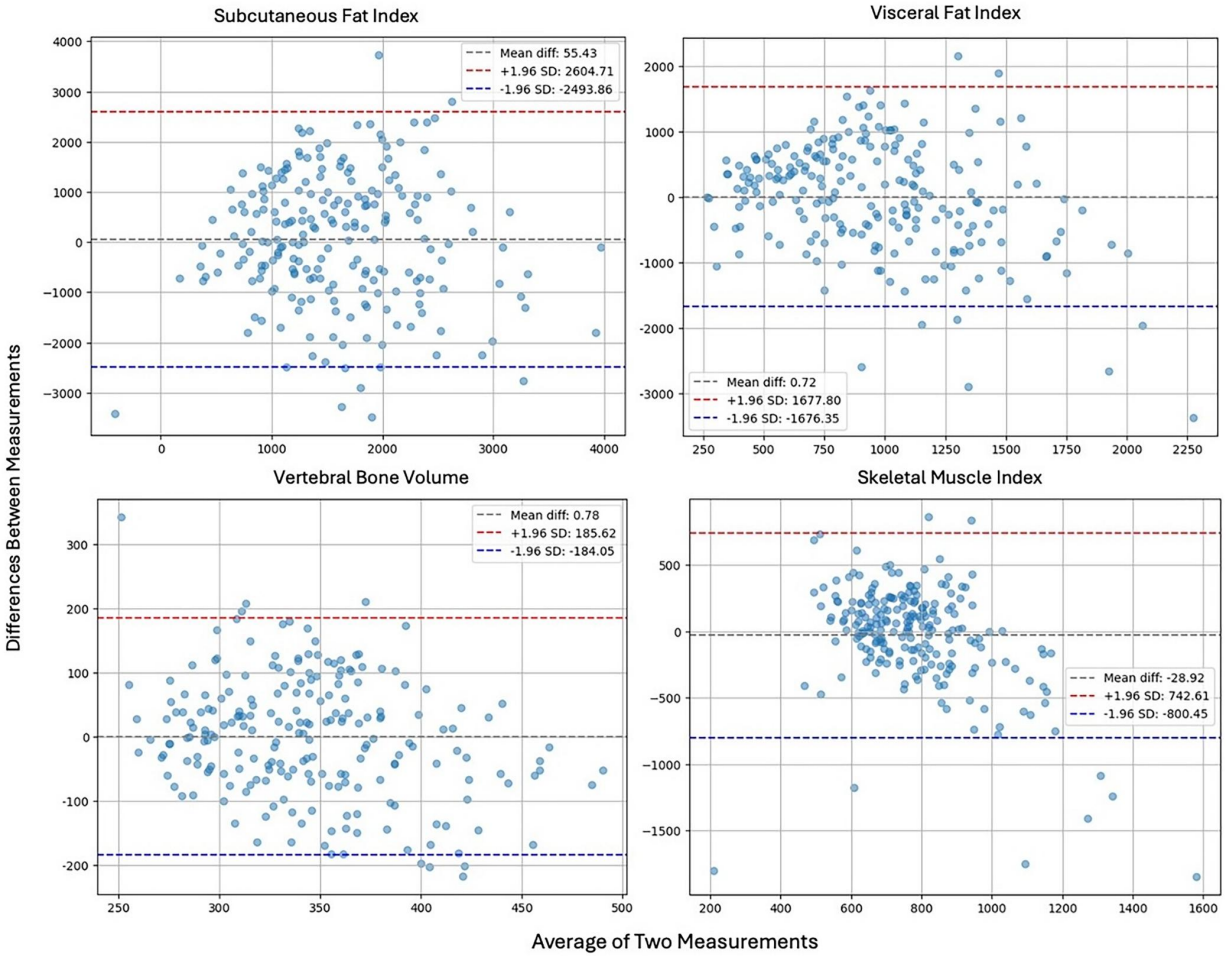
Bland-Altman plots showing the distribution of prediction errors with respect to ground truth (ie, values calculated from abdominal CT) for subcutaneous and visceral fat index, vertebral bone volume, and skeletal muscle volume.

The MAE of the best performing models for subcutaneous fat volume and visceral fat volume was 1054 cm^3^/m^2^ and 667 cm^3^/m^2^. MAE for vertebral bone volume was 75 cm^3^, and for skeletal muscle index was 267 cm^3^/m^2^. Bland-Altman plots for these body composition metrics can be seen in Figure 4.

### L3 slice level models

Our L3 level model achieved similar performance in the top-performing categories to the volumetric body composition (T12–L5) models. Its estimates on the hold-out test set achieved a correlation of 0.81 (0.76–0.85) for subcutaneous fat area and 0.74 (0.67–0.80) for visceral fat area.

### Explainability

**Figure 5.**
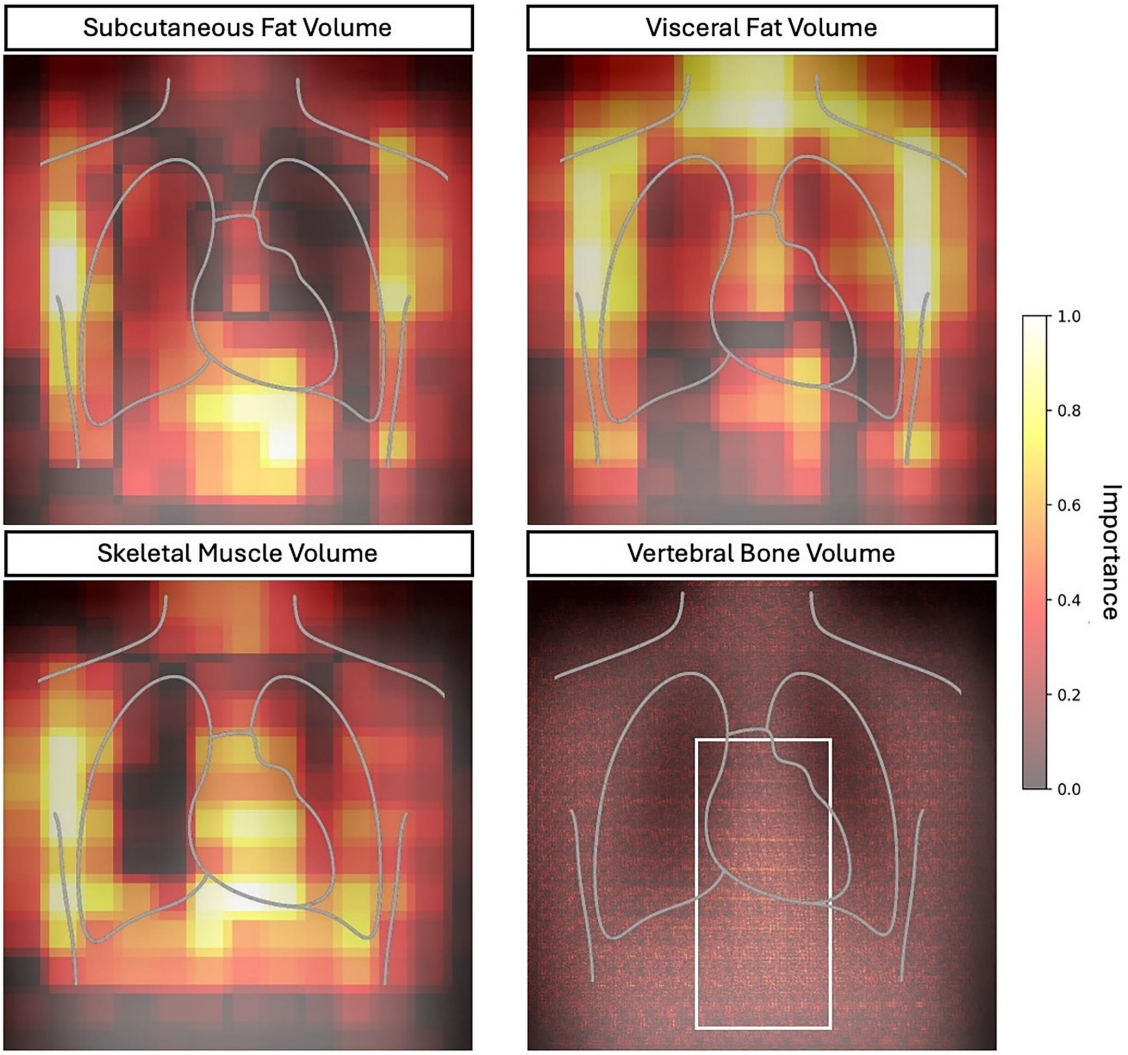
Saliency maps for feature importance in prediction of the top performing body composition metrics. For the first 3, occlusion-sensitivity saliency maps are depicted, whereas for the vertebral body volume, the saliency map is based on the integrated gradients method. The white box highlights the region of increased importance for vertebral body volume calculation that coincides with the region containing the lower thoracic and upper lumbar vertebral bodies. The images are aggregates of individual chest radiographs and their respective saliency maps in the hold-out test cohort.

**Figure 6.**
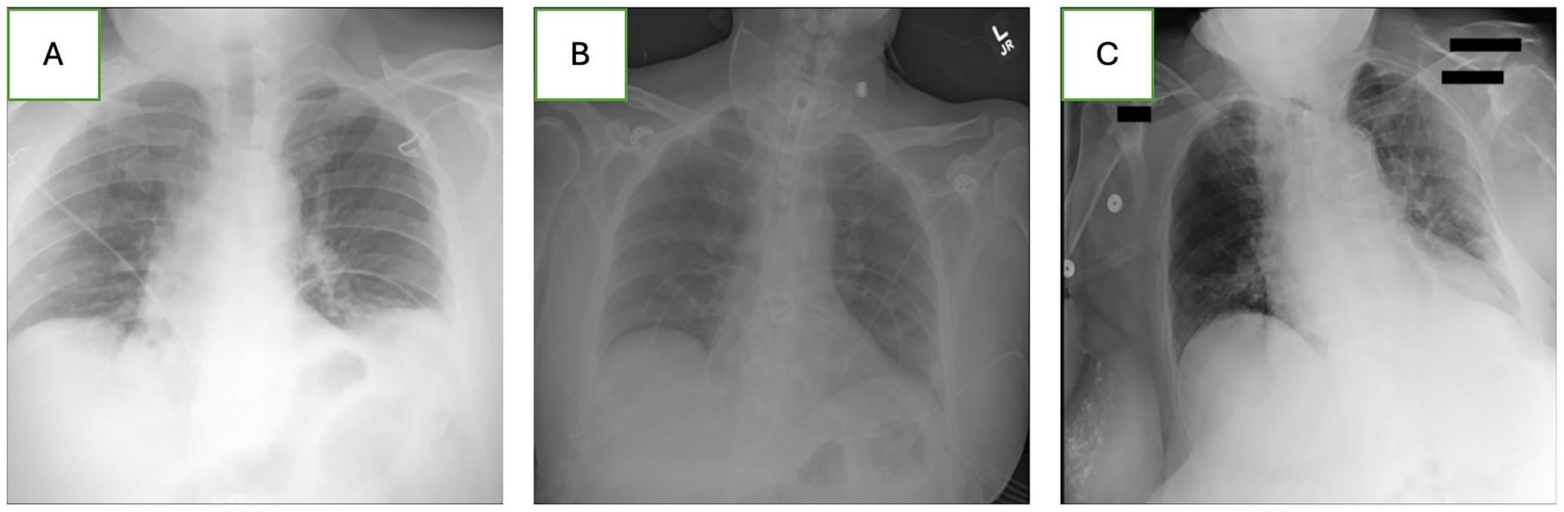
Three samples of the radiographs in which the model had poor performance. (A) Parts of the chest radiograph that are important to the model are missing from the image. eSFV vs SFV: 1525 cm^3^ vs 6320 cm^3^, eVFV vs VFV: 660 cm^3^ vs 4202 cm^3^, eSMI vs SMI: 150 cm^3^/m^2^ vs 1003 cm^3^/m^2^ B. This chest radiograph is dark and does not encompass some important regions for prediction. eSFV vs SFV: 6234 cm^3^ vs 1741 cm^3^, eVFV vs VFV: 3153 cm^3^ vs 5234 cm^3^, eSMI vs SMI: 929 cm^3^/m^2^ vs 1286 cm^3^/m^2^ C. The patient is rotated, and the lungs are not properly inflated. eSFV vs SFV: 4003 cm^3^ vs 5940 cm^3^, eVFV vs VFV: 2046 cm^3^ vs 3151 cm^3^, eSMI vs SMI: 2501 cm^3^/m^2^ vs 1050 cm^3^/m^2^. (e)SMI = (estimated) skeletal muscle index, (e)SFV = (estimated) subcutaneous fat volume, (e)VFV = (estimated) visceral fat volume.

The aggregate explainability saliency maps for the prediction of subcutaneous fat volume, visceral fat volume, vertebral bone volume, and skeletal muscle volume can be viewed in Figure 5. This figure shows that for the estimation of subcutaneous and visceral fat tissue volume, the model looks at the mediastinum (which contains visceral adipose tissue), the suprasternal region, and the sides of the trunk. Although the saliency maps overlap, they put different levels of importance on different parts. For example. The visceral fat volume model is more focused on the mediastinum and neck region compared to the subcutaneous fat volume model. The model for vertebral bone volume focuses on the part of the image that contains the lower thoracic vertebrae behind the mediastinum. A sample of 3 cases that resulted in poor performance in the best-performing model can be seen in Figure 6. These cases were selected from among the 6 cases with the worst performance, and each represents a distinct example of where our model may fail.

### Fairness analysis

We investigated the performance of our model within different age, sex, and BMI subgroups of our test cohort to identify potential biases in our model. In general, there was variability in model performance across various subgroups. Some observed trends include better performance in the estimation of subcutaneous fat volume in female individuals and those with higher BMI, and higher predictive performance for skeletal muscle and vertebral bone volume in younger patients. The results for the top-performing body composition metrics can be seen in the Supplementary Material Table S2.

## Discussion

In this study, we developed a multimodal deep learning model using chest radiographs and four clinical variables to estimate various body composition measures. Our model achieved good predictive performance in estimating subcutaneous adipose tissue, visceral adipose tissue, vertebral bone volume, and, to a lesser extent, skeletal muscle volume. Although these estimated metrics are less accurate compared with CT-based metrics, the resulting model can be used for a larger population of people to get estimated body composition metrics, which are more accurate surrogates compared to weight and BMI alone when CT scans or MRI are not available.

Our multimodal model outperformed unimodal clinical-only and imaging-only models, with performance increase being especially noticeable on estimating subcutaneous fat volume, visceral fat volume, and vertebral bone volume. The model achieved good performance in the estimation of skeletal muscle volume in training and validation sets. However, that performance was not replicated in the test set. Bland-Altman analysis demonstrated that the model had poor performance in predicting large skeletal muscle volume values. The combined model did not perform noticeably well on tissue-level body composition metrics or aortic calcification score levels. We also trained models to predict mid-L3 level body composition metrics. We showed that the models for mid-L3 body composition metrics perform similarly to the volumetric ones.

Our clinical model, which included sex at birth, weight, height, and age, could be used to estimate some body composition measures as well, especially subcutaneous fat area, skeletal muscle area, and aortic calcification score. However, a clinical model performs poorly when estimating other body composition metrics, especially visceral fat area. Visceral fat area is particularly important because it is correlated with adverse outcomes in various diseases.^3^^,^^5^ The addition of imaging data to clinical data resulted in a significant improvement in body composition estimation across most metrics.

The ability to differentiate subcutaneous and visceral fat is specifically important because of the importance of visceral fat volume in the prediction of various risk factors from cardiometabolic disease to cancer.^3^^,^^6^ Another important body composition metric, which is proven to be a risk factor for adverse outcomes, is sarcopenia.^23^ We looked at both skeletal muscle volume, radiodensity, and intramuscular fat in our study. Although our final model only achieved moderate correlation in predicting skeletal muscle volume, during training and validation, we observed more promising performance. This decline in performance could be attributed to overfitting of the model or could be due to other differences between the training and testing cohorts. In addition, our model had moderate to good performance in estimating the muscle radiodensity (*r* = 0.69, 0.61–0.75). Additional work is required to explore the possibility of estimating these 2 metrics from radiographs.

Previous studies that investigated the timing of fusion for medical data have found conflicting answers. We found that although the performance of late- and intermediate-based fusion models was very close, the late fusion model had a higher performance. These differences show the importance of experimenting with different fusion strategies in multimodal deep learning studies. Our findings indicate that it would be beneficial to use the optimum fusion strategy for each body composition metric during inference. Refer to Supplementary Material Section 5 for further discussion.

The saliency maps show that the model is looking into the appropriate regions of the radiograph when estimating body composition metrics, including the mediastinum. Our failure analysis reveals that compared to a typical chest radiograph, cases with low performance demonstrate problems like incomplete coverage of the trunk on both sides, patient rotation, inadequate lung inflation, and low exposure. Based on these findings, we hypothesize that our model’s performance could be higher if provided with high-quality chest radiographs.

Although we achieved good performance on multiple tasks in this study, the sample size of our study was relatively small for this task. Another limitation of our study is the use of abdominal CT scan-based body composition metrics. Use of whole-body CT scans or use of quantitative CT scans can help generate more accurate ground truth values. In addition, future efforts should involve strategies to measure certainty around estimates and improve robustness with respect to artifacts. Finally, vision transformers have shown great promise in deep learning applications in radiology and should be experimented with as an alternative to CNN’s future.

In conclusion, we developed a multitask, multimodal, deep learning model to estimate various body composition metrics, including subcutaneous fat volume, visceral fat volume, and vertebral bone volume, using a chest radiograph and 4 relevant clinical variables. We also explored how various strategies for fusion can contribute to varying model performance.

## Supplementary Material

umaf035_Supplementary_Data

## Data Availability

All data used in this study can be accessed by researchers through a paid subscription at http://studio.truveta.com/.
